# Calcium-Sensing Receptor Contributes to Hyperoxia Effects on Human Fetal Airway Smooth Muscle

**DOI:** 10.3389/fphys.2021.585895

**Published:** 2021-03-15

**Authors:** Anne M. Roesler, Jovanka Ravix, Colleen M. Bartman, Brijeshkumar S. Patel, Marta Schiliro, Benjamin Roos, Lisa Nesbitt, Christina M. Pabelick, Richard J. Martin, Peter M. MacFarlane, Y. S. Prakash

**Affiliations:** ^1^Department of Anesthesiology and Perioperative Medicine, Mayo Clinic, Rochester, MN, United States; ^2^Department Physiology and Biomedical Engineering, Mayo Clinic, Rochester, MN, United States; ^3^Department of Pediatrics, Case Western Reserve University, Cleveland, OH, United States

**Keywords:** fetal airway, oxygen, calcium, smooth muscle contractility and remodeling, Class C GPCR

## Abstract

Supplemental O_2_ (hyperoxia), necessary for maintenance of oxygenation in premature infants, contributes to neonatal and pediatric airway diseases including asthma. Airway smooth muscle (ASM) is a key resident cell type, responding to hyperoxia with increased contractility and remodeling [proliferation, extracellular matrix (ECM) production], making the mechanisms underlying hyperoxia effects on ASM significant. Recognizing that fetal lungs experience a higher extracellular Ca^2+^ ([Ca^2+^]_o_) environment, we previously reported that the calcium sensing receptor (CaSR) is expressed and functional in human fetal ASM (fASM). In this study, using fASM cells from 18 to 22 week human fetal lungs, we tested the hypothesis that CaSR contributes to hyperoxia effects on developing ASM. Moderate hyperoxia (50% O_2_) increased fASM CaSR expression. Fluorescence [Ca^2+^]_i_ imaging showed hyperoxia increased [Ca^2+^]_i_ responses to histamine that was more sensitive to altered [Ca^2+^]_o_, and promoted IP_3_ induced intracellular Ca^2+^ release and store-operated Ca^2+^ entry: effects blunted by the calcilytic NPS2143. Hyperoxia did not significantly increase mitochondrial calcium which was regulated by CaSR irrespective of oxygen levels. Separately, fASM cell proliferation and ECM deposition (collagens but not fibronectin) showed sensitivity to [Ca^2+^]_o_ that was enhanced by hyperoxia, but blunted by NPS2143. Effects of hyperoxia involved p42/44 ERK *via* CaSR and HIF1α. These results demonstrate functional CaSR in developing ASM that contributes to hyperoxia-induced contractility and remodeling that may be relevant to perinatal airway disease.

## Introduction

Supplemental O_2_ (hyperoxia) even at moderate levels (≤ 50% O_2_) is an unfortunately necessary intervention in the context of premature birth to maintain oxygenation and ensure survival. However, prolonged hyperoxia is also recognized as contributing to pediatric airway diseases such as wheezing and asthma that persist beyond the neonatal period (Jobe, 2011; Britt et al., 2013; Been et al., 2014; Gough et al., 2014; Vollsaeter et al., 2015; Landry et al., 2016). In this regard, airway smooth muscle (ASM) is a key cell type involved in hypercontractility and remodeling (Prakash, 2013, 2016; Thompson et al., 2015; Faksh et al., 2016; Vogel et al., 2017), responding to hyperoxia via increased intracellular calcium ([Ca^2+^]_i_), cell proliferation, and extracellular matrix (ECM) deposition (Hartman et al., 2012; Vogel et al., 2017). Such effects are also noted in newborn mouse models of moderate hyperoxia exposure that show persistent increases in bronchial wall diameter and enhanced responses to methacholine challenge (Wang et al., 2014; Onugha et al., 2015). While these data support a role for neonatal hyperoxia in airway disease, the mechanisms underlying hyperoxia effects are still under exploration.

We have previously demonstrated that moderate hyperoxia (50% O_2_) enhances [Ca^2+^]_i_) regulation in human fetal ASM (fASM) from lungs of 18–22 week fetuses, a developmental period of rapid bronchial growth and proximate to neonatal survival in the ICU. However, compared to adult, the mechanisms of [Ca^2+^]_i_ regulation and of O_2_ effects are less-studied. We previously characterized the suitability of isolated fASM cells in exploring regulation of [Ca^2+^]_i_ (Hartman et al., 2012), showing that fASM cells express regulatory proteins and [Ca^2+^]_i_ responses similar to that of adult ASM. But the mechanisms of hyperoxia effects in fASM are not well-known. In this regard, an important aspect of fetal development is that the extracellular environment that regulates airway growth shows higher extracellular Ca^2+^ ([Ca^2+^]_o_) level of ~1.7 mM compared to adult (~1.2 mM) (Kovacs and Kronenberg, 1997; Riccardi et al., 2013). Accordingly, it is possible that [Ca^2+^]_o_ itself contributes to ASM structure and function, and could further mediate or modulate hyperoxia effects. [Ca^2+^]_o_ is normally sensed by the Class C GPCR family member Ca^2+^ sensing receptor (CaSR). The CaSR is well-recognized for its role in the parathyroid, kidney and bone toward regulation of body calcium levels (Brennan et al., 1833; Riccardi and Kemp, 2012; Goltzman and Hendy, 2015). For example, calcimimetics (CaSR activators) are used to treat hyperparathyroidism while negative allosteric modulators (calcilytics, e.g., NPS2143) target genetic hypocalcemia disorders (Hannan et al., 2017; Nemeth et al., 2018). We and others have contributed to understanding CaSR in non-calciotropic tissues where it can regulate several cellular functions such as [Ca^2+^]_i_, gene expression, proliferation and ECM (Brennan et al., 1833; Riccardi and Kemp, 2012; Riccardi et al., 2013; Yarova et al., 2015; Schepelmann et al., 2016) all relevant to ASM. Indeed, we previously showed that functional CaSR is present in adult human ASM and contributes to airway hyperreactivity in the context of asthma (Yarova et al., 2015). CaSR is expressed in embryonic lung mesenchyme and can modulate branching morphogenesis (Riccardi et al., 2013; Brennan et al., 2016). Recently, we showed that human fASM expresses CaSR and can regulate [Ca^2+^]_i_ in response to [Ca^2+^]_o_ in the context of ASM contractility, and promotes cell proliferation (Roesler et al., 2019). Accordingly, in the present study, we explored the hypothesis that the CaSR contributes to hyperoxia effects on fASM in the context of enhanced contractility and remodeling.

## Materials and Methods

### Cell Culture

Human fASM cells were isolated as previously described (Hartman et al., 2012; Vogel et al., 2015, 2017; Roesler et al., 2019). Tracheobronchial samples of de-identified 18–22 week fetuses following demise (StemCell Express, Arlington, MA) were denuded of epithelium and enzymatically digested. Isolated cells were cultured in Dulbecco's modified Eagle's medium/F12 (Life Technologies, Rockville, MD) supplemented with 10% fetal bovine serum (FBS), penicillin, and streptomycin (Life Technologies). Sub-culturing was limited to 10 passages while experiments, performed in serum-starved conditions (0.5% FBS), were limited to passage 5. The study was considered exempt by the Mayo Institutional Review Board since maternal or fetal identifiers were not available and the isolated cells were stored with unique identifiers unrelated to their source.

### Oxygen Exposure

Cells were grown under standard conditions with 21% O_2_ or 50% O_2_ [considered hyperoxia relative to standard cell culture conditions of 21% O_2_ and also the fetal environment that is relatively hypoxic (Vogel et al., 2015)] for 48 h under serum-starved conditions. Experiments were then performed for immunofluorescence, [Ca^2+^]_i_ imaging, proliferation, ECM production or signaling.

### Immunofluorescence

Standard techniques were applied (Roesler et al., 2019). fASM cells were fixed with 4% paraformaldehyde for 10 min and then immunostained with CaSR antibody (Invitrogen #PA1-934A, rabbit anti-CaSR, 1:100 dilution; Abcam #150074, Alexa555 donkey anti-rabbit IgG, 1:500). Nuclei were counterstained using DAPI. Omission of primary antibodies was used as a negative staining control.

### [Ca^2+^]_i_ Imaging

Previously described techniques for fura-2 based fASM [Ca^2+^]_i_ imaging were used (Roesler et al., 2019). fASM cells were grown to 50% confluence in 8-well glass-bottomed imaging chambers (Thermo Fisher Scientific, Scotts Valley, CA). Experiments were done in Hanks Balanced Salt Solution (HBSS) where [Ca^2+^]_o_ could be varied. As the intent was to explore the CaSR, which is sensitive to [Ca^2+^]_o_, cells were deprived of Ca^2+^ for 12 h before experimentation. Cells were then treated for 1 h with either medium (vehicle) or 1 μM NPS2143 (calcilytic Tocris) (Yarova et al., 2015; Roesler et al., 2019). Cells were loaded with 5 μM fura-2/AM for 30 min in 0 mM [Ca^2+^]_o_ and then washed. Procedures were done in separate cell sets exposed to the same [Ca^2+^]_o_ throughout: 0, 0.5, 1, or 2 mM Ca^2+^. Imaging involved an inverted microscope (Nikon Eclipse Ti-U) with perfusion used to alter [Ca^2+^]_o_ or add agonists as necessary. Baseline, peak, and amplitude of [Ca^2+^]_i_ responses were recorded and analyzed.

### Western Blots

Standard techniques were used. Total protein content was measured using DC Protein Assay kit (BioRad) and a minimum of 25 μg equivalent protein loaded in 4–15% gradient gels (Criterion Gel System; Bio-Rad), followed by transfer to nitrocellulose membranes (Bio-Rad Trans-Blot Turbo), blocking with 5% BSA, and overnight exposure to primary antibodies. Bands were detected on Li-Cor Odyssey system using LiCOR near-red secondary antibodies. Densitometric analysis was performed using Image Studio software.

### Cell Proliferation

fASM cell proliferation was assessed using a MTS assay as previously described (Parikh et al., 2018; You et al., 2019). Additionally, Western blot for PCNA was used as a marker for proliferation (Abcam #13110, rabbit anti-PCNA; 1: 1000).

### ECM Deposition

fASM deposition of ECM was assessed using a modified In-Cell Western technique (Freeman et al., 2017). Briefly, cells at 50% confluence in 96-well plates were exposed to 21 or 50% O_2_ for 72 h. Cells were then lysed with NH_4_OH, and the deposited ECM immunostained with primary antibody of interest. Targets were collagen I (Abcam #ab34710, rabbit anti-collagen I, 1:200), collagen III (Abcam #ab7778, rabbit anti-collagen III, 1:200) and fibronectin (Abcam #ab2413, rabbit anti-fibronectin, 1:200). Plates were incubated in far red-conjugated secondary antibodies and quantified *via* densitometry on a Li-Cor OdysseyXL imaging system. Blank wells lacking cells were treated with media and all primary and secondary antibodies to control for any background signal. Additionally, antibody specificity has been previously verified (Freeman et al., 2017) along with positive controls such as native human ECM proteins from the same vendor.

### Mechanisms of Hyperoxia Effects

Based on initial observations in the study on altered [Ca^2+^]_i_ regulation as well as proliferation or ECM in fASM with 50% O_2_, we explored potential mechanisms involving CaSR. In this study, toward regulation of [Ca^2+^]_i_ we focused on (1) Endoplasmic reticulum (ER) Ca^2+^ release involving IP_3_ receptors (Yarova et al., 2015; Roesler et al., 2019) testing the effect of 20 μM Xestospongin C, an IP_3_ receptor blocker (Yarova et al., 2015), on [Ca^2+^]_i_ responses to histamine in the presence of the CaSR blocker NPS2143; (2) Whether CaSR is involved in modulating mitochondrial calcium using the fluorescent indicator rhod-2/AM (2.5 μM) (Delmotte et al., 2012) in cells exposed to 50% O_2_ with/without the CaSR inhibitor NPS2143; (3) Using Western blots, signaling intermediates relevant to CaSR signaling as shown in adult ASM (Delmotte et al., 2012), specifically p38 and p42/44 MAP kinases (Cell Signaling #4511 rabbit anti-phospho-p38, #8690 rabbit anti-p38, #9102, #4377 rabbit anti-phospho-ERK1/2 rabbit anti-ERK1/2; all at 1:5000); (4) Potential role of HIF1α using the hypoxia mimetic CoCl_2_ (100 μM) in the presence vs. absence of NPS2143 in addition to the 21 and 50% exposures. Changes in nuclear to cytosolic HIF1α ratio were explored using subcellular fractionation (Thermo Scientific, #78840) and quantitative Western blot (automated Simple Western by Protein Simple; Cell Signaling #36169, rabbit anti-HIF1α, 1:100; Abcam #ab63766, rabbit TBP (TATA-binding protein) nuclear fraction loading control, 1:100; Cell Signaling #2118, rabbit, GAPDH cytoplasmic fraction loading control, 1:400).

### Statistics

All experiments were performed in fASM cells from four or more fetuses. For cell imaging studies, at least 20 cells/protocol/sample were sampled. “N” values represents numbers of samples. Proliferation experiments were repeated at least three times for each sample. All data are expressed as mean ± SD. Statistical analysis was performed using individual Student *t*-test, ANOVA, or mixed-effects analysis with Tukey's correction for multiple comparisons. Statistical significance was tested at the *p* < 0.05 level. Statistical analyses were performed using GraphPad Prism 7.03 software.

## Results

### CaSR in Human fASM

**Figure 1:**
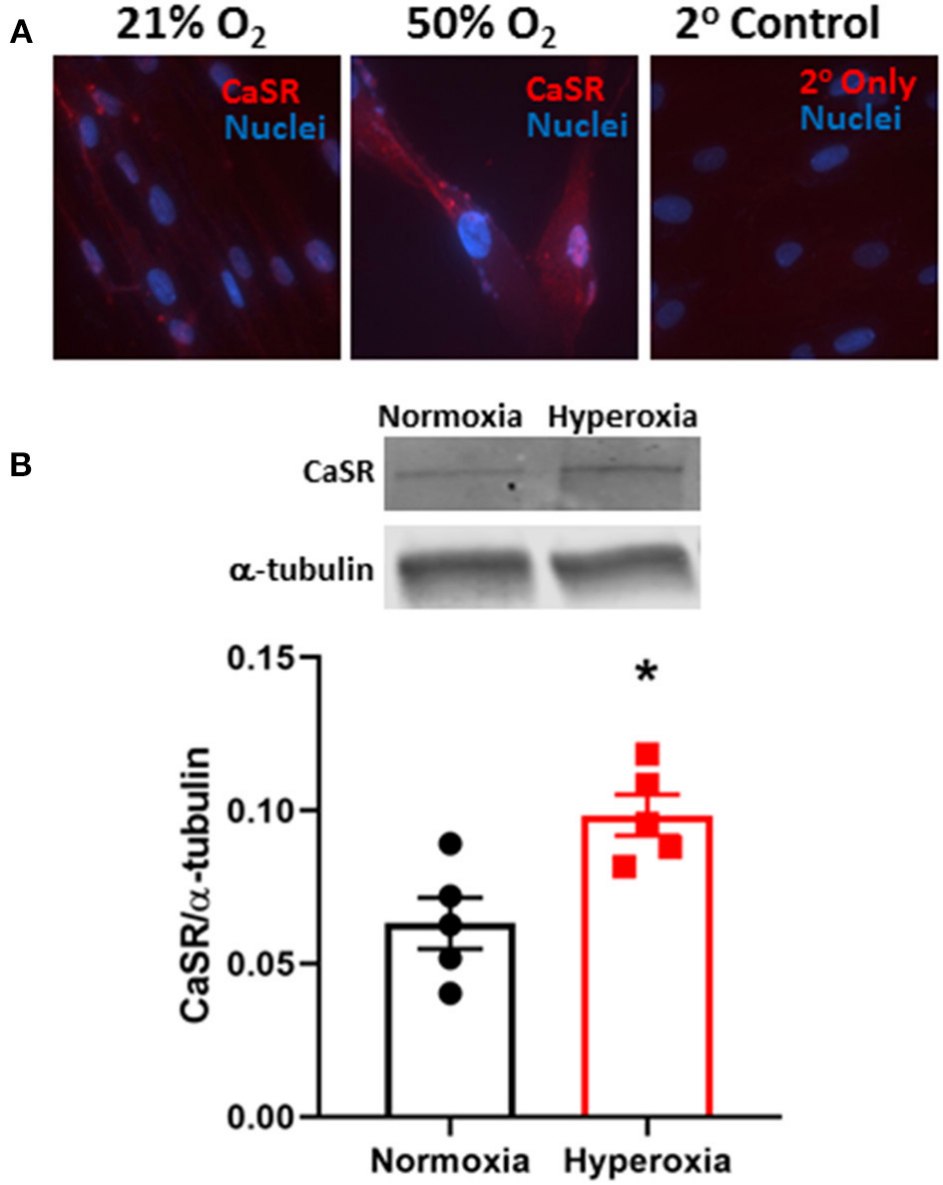
Hyperoxia effect on expression of the extracellular Ca^2+^ ([Ca^2+^]_o_) sensing receptor (CaSR) in developing human fetal (<22 week gestation) airway smooth muscle (fASM) cells. **(A)** Immunofluorescence staining for CaSR (red) showed increased expression with 50% O_2_ exposure for 48 h (nuclei stained with DAPI, blue) compared to 21% O_2_ (normoxia). Representative of *N* = 4. **(B)** Western blot analysis confirmed increased CaSR expression in fASM cells with hyperoxia. *N* = 5. Values are means + SD. *indicates significant hyperoxia effect (*p* < 0.05).

We previously showed that CaSR was localized to smooth muscle layers of airways in human fetuses <22 week and that isolated fASM cells express CaSR (Roesler et al., 2019). In fASM cells, exposure to 50% O_2_ for 48 h increased immunofluorescence staining for CaSR (Figure 1). Western blot of fASM lysates showed significant increase in CaSR expression with 50% O_2_ (Figure 1; *p* < 0.05).

### Hyperoxia and [Ca^2+^]i

**Figure 2:**
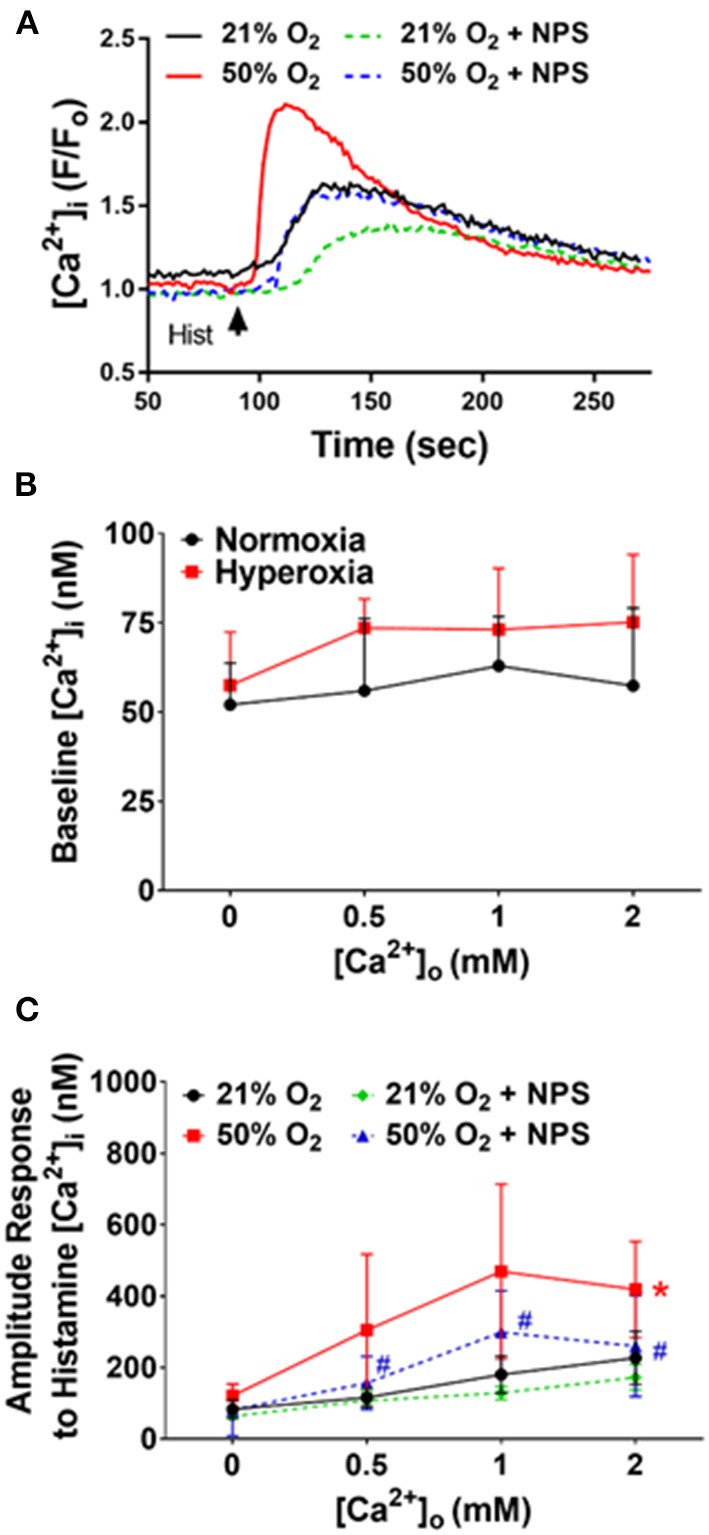
Hyperoxia, CaSR and fASM intracellular Ca^2+^ ([Ca^2+^]_i_). **(A)** In fura-2 loaded fASM cells, hyperoxia increased [Ca^2+^]_i_ responses to 10 uM histamine compared to normoxia. Such effects were blunted by the CaSR antagonists NPS2143. **(B)** Increasing [Ca^2+^]_o_ to modulate CaSR activity slightly increased hyperoxia effects on baseline [Ca^2+^]_i_ but not significantly. **(C)** In contrast, hyperoxia substantially increased the amplitude of [Ca^2+^]_i_ responses, with increased sensitivity to [Ca^2+^]_o_: effects inhibited by NPS2143. *N* = 4–5; Means + SD. *indicates significant hyperoxia effect, ^#^significant NPS2143 effect (*p* < 0.05).

We previously showed that [Ca^2+^]_i_ in fASM cells increases in response to agonists such as ACh and histamine (Hartman et al., 2012; Roesler et al., 2019). We recently found that such [Ca^2+^]_i_ responses are sensitive to increasing [Ca^2+^]_o_ and modulated by CaSR agonist R568 or the antagonist NPS2143 (Roesler et al., 2019). Here, we found that after 48 h exposure to 50% O_2_, baseline [Ca^2+^]_i_ levels did not significantly change compared to 21% O_2_, and while they showed small increases with increasing [Ca^2+^]_o_ (0–2 mM Ca^2+^), baseline levels were not different between the two groups (Figure 2). However, 48 h of 50% O_2_ significantly enhanced [Ca^2+^]_i_ responses to 10 μM histamine (Figure 2; *p* < 0.05). Here, cells in normoxia showed increasing [Ca^2+^]_i_ responses with higher [Ca^2+^]_o_ levels. This [Ca^2+^]_o_ sensitivity was substantially increased in 50% O_2_-exposed cells. The contribution of CaSR to this increased sensitivity to [Ca^2+^]_o_ was shown by the significant inhibitory effect of NPS2143 (Figure 2; *p* < 0.05).

**Figure 3:**
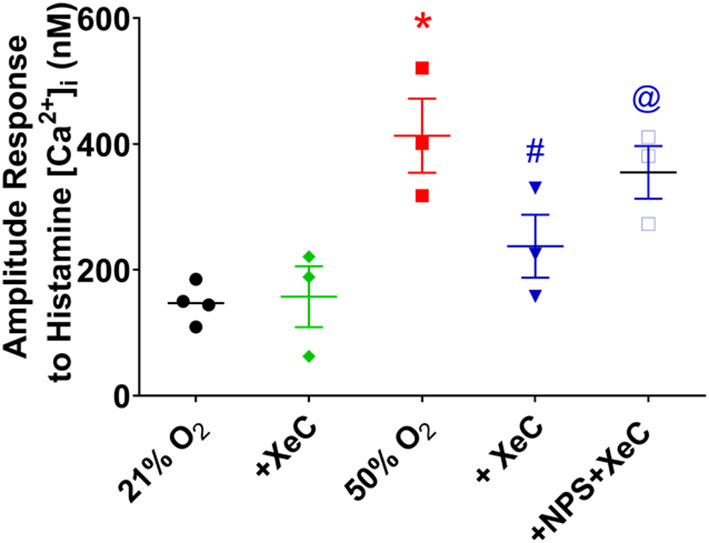
Hyperoxia and intracellular Ca^2+^ release. The CaSR is known to activate the PLC/IP_3_ pathway. Hyperoxia-enhanced [Ca^2+^]_i_ responses to histamine were significantly blunted by the IP_3_ receptor inhibitor Xestospongin C (XeC). *N* = 4–5. Means ± SD. *indicates significant hyperoxia effect, ^#^significant NPS2143 effect, @indicates significant XeC effect (*p* < 0.05).

In adult as well as fetal ASM, we previously showed that CaSR enhances [Ca^2+^]_i_ *via* endoplasmic reticulum (ER) Ca^2+^ release involving IP_3_ receptors (Yarova et al., 2015; Roesler et al., 2019). In fASM cells exposed to 21% O_2_, pre-exposure to 20 μM of the IP_3_ receptor blocker Xestospongin C for 30 min significantly blunted [Ca^2+^]_i_ responses to histamine (Figure 3; *p* < 0.05). In cells exposed to 50% O_2_ that showed higher [Ca^2+^]_i_ responses, Xestospongin C had greater inhibitory effects (Figure 3; *p* < 0.05). In the presence of the CaSR blocker NPS2143, Xestospongin C effects were reduced (Figure 3).

**Figure 4:**
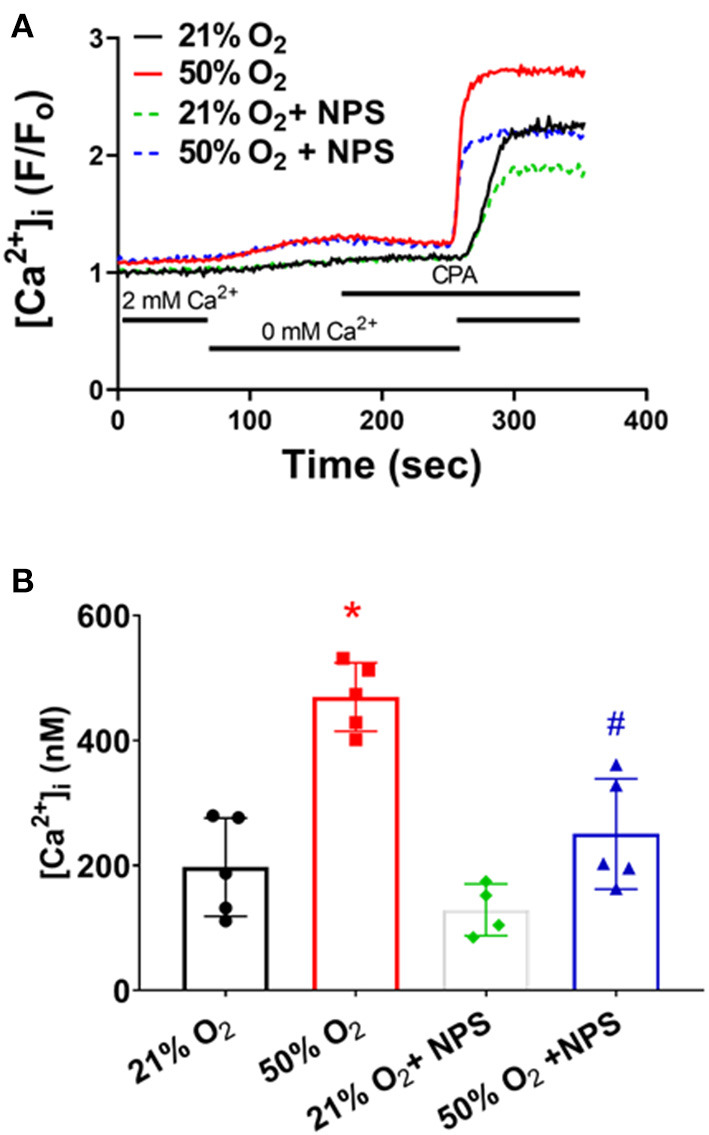
Hyperoxia, CaSR and store-operated Ca^2+^ entry (SOCE). **(A)** Following removal of [Ca^2+^]_o_, depletion of intracellular Ca^2+^ stores using the sarcoplasmic reticulum reuptake inhibitor cyclopiazonic acid (CPA) caused an expected increase in [Ca^2+^]_i_. Subsequent rapid reintroduction of 2 mM [Ca^2+^]_o_ triggered SOCE that was substantially enhanced in hyperoxia-exposed cells, but suppressed by NPS. **(B)** Summary of hyperoxia and NPS effects on SOCE. *N* = 5 for summaries; Means + SD. *indicates significant hyperoxia effect, ^#^significant NPS2143 effect (*p* < 0.05).

Given their proliferative tendency, fASM cells are more reliant on plasma membrane Ca^2+^ influx mechanisms, including store-operated Ca^2+^ entry (SOCE) induced by sarcoplasmic reticulum Ca^2+^ depletion (Hartman et al., 2012). Depletion of SR Ca^2+^ using the SERCA inhibitor cyclopiazonic acid in the absence of [Ca^2+^]_o_ followed by rapid reintroduction of [Ca^2+^]_o_ (with 1 μM nifedipine to block L-type Ca^2+^ channels) is used to assess [Ca^2+^]_i_ reflecting SOCE. fASM cells exposed to 50% O_2_ showed significantly higher SOCE (Figure 4; *p* < 0.05) which was substantially blunted by NPS2143 (Figure 4; *p* < 0.05).

**Figure 5:**
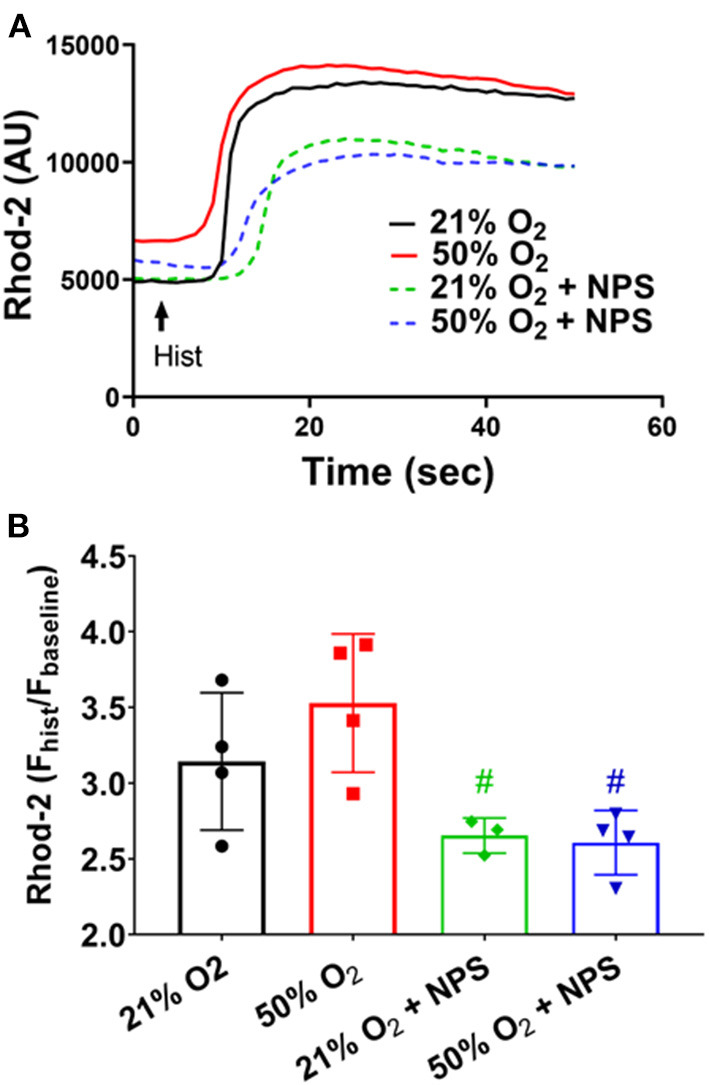
Hyperoxia, CaSR and mitochondrial Ca^2+^. In rhod-2 loaded fASM cells, histamine exposure resulted in slower increases in mitochondrial Ca^2+^. **(A)** 50% O_2_ did not significantly influence mitochondrial Ca^2+^ responses to histamine **(B)** However, NPS2143 did significantly alter such responses in both 21 and 50% O_2_ exposed cells. *N* = 4–5. Means ± SD. ^#^significant NPS2143 effect (*p* < 0.05).

Separately, given that hyperoxia can influence mitochondrial structure (Hartman et al., 2012), we explored whether CaSR is involved in altering mitochondrial calcium using the fluorescent indicator rhod-2/AM (2.5 uM) (Delmotte et al., 2012) in cells exposed to 50% O_2_ with/without the CaSR inhibitor NPS2143. Interestingly, in rhod-2 loaded fASM cells, 50% O_2_ did not significantly influence mitochondrial Ca^2+^ responses to histamine (Figure 5). However, NPS2143 did significantly alter such responses in both 21 and 50% O_2_ exposed cells (Figure 5).

### Hyperoxia and Remodeling

**Figure 6:**
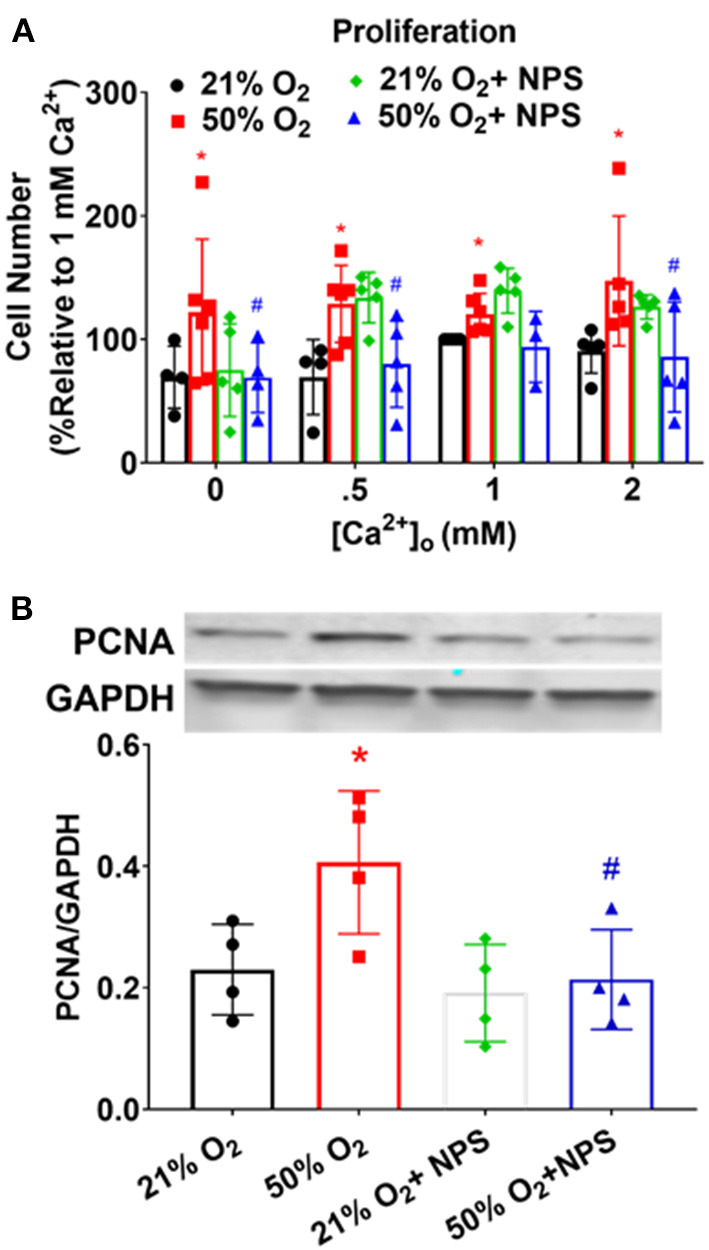
Hyperoxia, CaSR, and fASM cell proliferation. **(A)** Increasing [Ca^2+^]_o_ enhanced fASM proliferation over a 48 h period: effects enhanced by 50% O_2_. Inhibiting CaSR with NPS blunted hyperoxia effects on proliferation. *N* = 4–5. **(B)** Proliferation effects measured by MTS assay in **(A)** were verified by changes in the marker PCNA. Means ± SD. *indicates significant hyperoxia effect, ^#^significant NPS2143 effect (*p* < 0.05).

Proliferation of human fASM cells (Hartman et al., 2012; Martin et al., 2015; Vogel et al., 2017) is sensitive to [Ca^2+^]_o_ and to CaSR (Roesler et al., 2019). Exposure to 50% O_2_ significantly increased fASM proliferation at different [Ca^2+^]_o_ levels while NPS2143 significantly blunted hyperoxia effects (Figure 6; *p* < 0.05). These data using MTS assay were corroborated by Western blots for PCNA (Figure 6).

**Figure 7:**
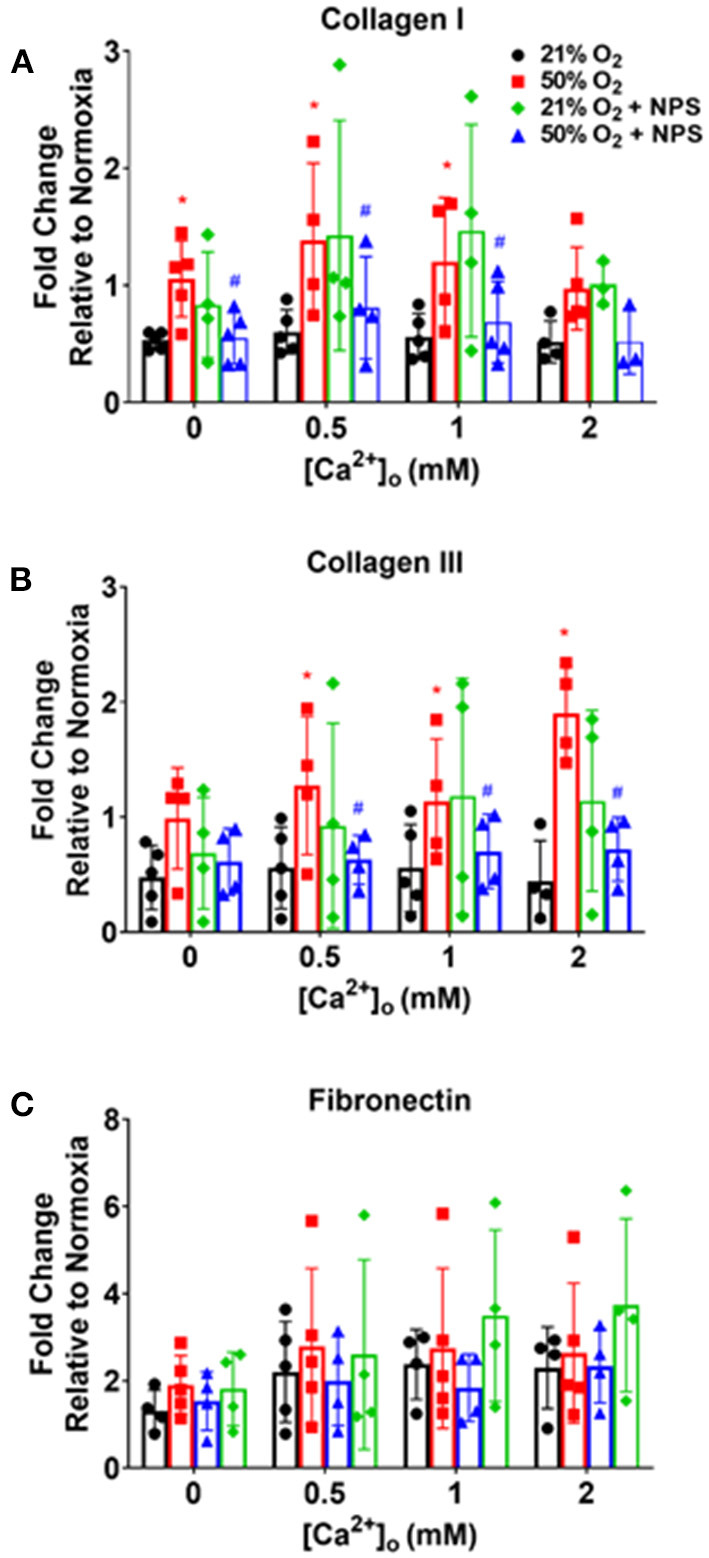
Hyperoxia, CaSR and fASM extracellular matrix (ECM) deposition. Hyperoxia increased fASM deposition of collagen I **(A)**, collagen III **(B)** and fibronectin **(C)** but showed differential responsiveness to [Ca^2+^]_o_. Regardless, NPS blunted hyperoxia effects on the collagens but not fibronectin. *N* = 4–5. Means + SD. *indicates significant hyperoxia effect, ^#^significant NPS2143 effect (*p* < 0.05).

Exposure of fASM to 50% O_2_ increased deposition of collagen I and collagen III (Figure 7; *p* < 0.05) but interestingly not of fibronectin. Hyperoxia effects on these ECM proteins showed some sensitivity to [Ca^2+^]_o_ but was not consistent between the collagens or to specific [Ca^2+^]_o_ levels. Nonetheless, NPS2143 significantly blunted hyperoxia effects on these ECM proteins (Figure 7; *p* < 0.05).

### Mechanisms of Hyperoxia Effects

**Figure 8:**
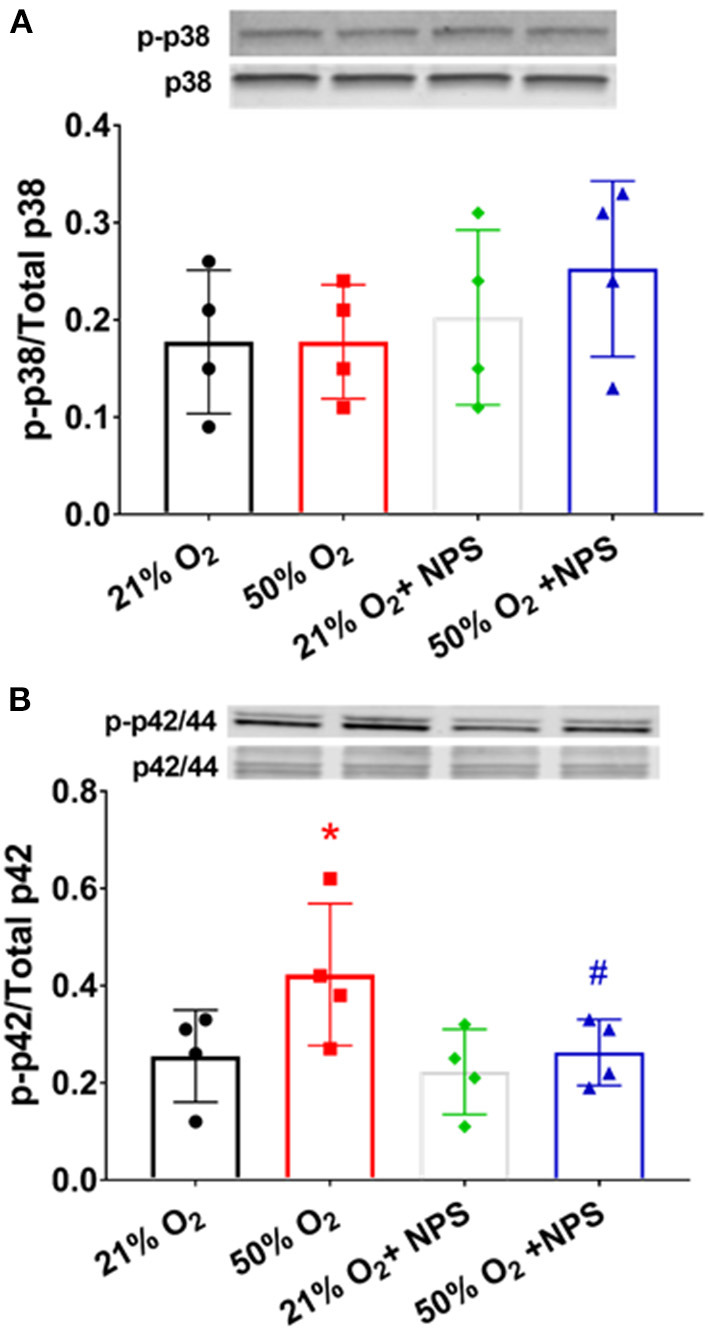
Mechanisms of hyperoxia effect. Exposure to 50% O_2_ did not alter p38 MAP kinase phosphorylation **(A)** but did increase p42/44 MAP kinase phosphorylation **(B)** NP2143 blunted hyperoxia effects on p42/44 MAP kinase phosphorylation. *N* = 3–4. Means ± SD. *indicates significant hyperoxia effect, ^#^significant NPS2143 effect (*p* < 0.05).

Exposure to 50% O_2_ increased p42/44 MAP kinase phosphorylation (Figure 8; *p* < 0.05) but did not alter p38 phosphorylation. NP2143 blunted hyperoxia effects on p42/44 MAP kinase phosphorylation (*p* < 0.05).

**Figure 9:**
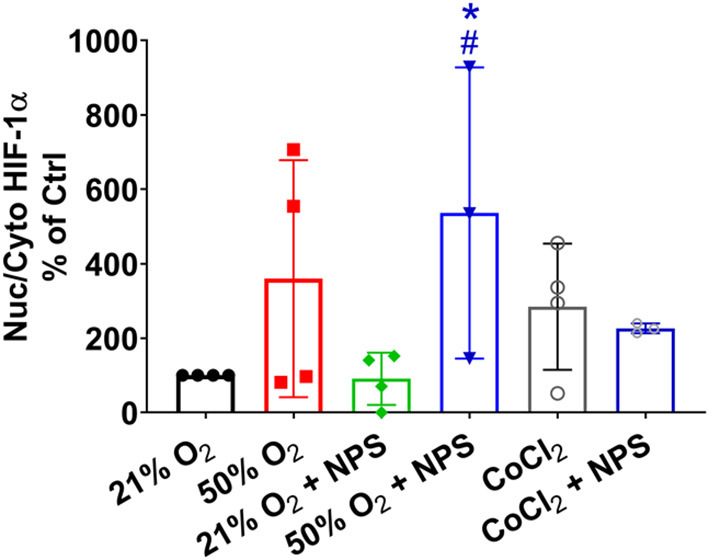
Hyperoxia and HIF1α. Similar to the hypoxia mimetic CoCl_2_, exposure to 50% O_2_ increased nuclear levels of HIF1α albeit with substantial variability. Pre-exposure to NPS2143 increased nuclear-cytosolic HIF1α in 50% O_2_ compared to 21% O_2_ (with or without NPS2143), but did not significantly reduce CoCl_2_ effects. *N* = 3–4. Means ± SD. *indicates significant effect of NPS2143 in hyperoxia vs. normoxia; ^#^indicates significant effect of NPS2143 in hyperoxia vs. NPS2143 in normoxia (*p* < 0.05).

Interestingly, previous studies (Wang et al., 2018; Noble et al., 2019; Wang and Noble, 2020) have found that hypoxia exposure results in altered ASM phenotypes similar to that with hyperoxia including ASM thickness and contractility, at least in mouse models. While it is not clear how hyperoxia and hypoxia can lead to similar physiological changes, we explored HIF1α in our models. In experiments intended to explore comparisons between hypoxia and hyperoxia, nuclear-cytosolic ratios of HIF1α were increased by CoCl_2_ compared to 21% O_2_, consistent with being a hypoxia mimetic (Figure 9). Exposure to 50% O_2_ also increased nuclear levels of HIF1α although there was variability in the extent of change (Figure 9). Pre-exposure to NPS2143 increased hyperoxia effects on nuclear-cytosolic HIF1α levels compared to 21% O_2_ regardless of pre-exposure to NPS2143 (Figure 9; *p* < 0.05). Additionally, NPS2143 did not substantially alter nuclear-cytosolic HIF1α levels in the CoCl_2_ condition (Figure 9).

## Discussion

Our study demonstrates that in developing human ASM, a functional CaSR is involved in the effects of moderate hyperoxia (50% O_2_) on [Ca^2+^]_i_ responses to bronchoconstrictor agonist, cellular proliferation and deposition of ECM in the context of airway hyperreactivity and remodeling. Data showing functional CaSR in fASM is consistent with our previous reports (Yarova et al., 2015; Roesler et al., 2019) and that in pulmonary artery (Tang et al., 2016; Xiao et al., 2017). Relevance of CaSR in developing ASM lies in its response to the known higher, physiologically relevant [Ca^2+^]_o_ concentrations in fetal lung (Kovacs and Kronenberg, 1997; Riccardi et al., 2013), its ability to elevate fASM [Ca^2+^]_i_ and contractility as we previously showed (Roesler et al., 2019) and the contribution of airway contractility to lung growth (Jesudason, 2009). Relevance of CaSR in hyperoxia-exposed fASM lies in the potential to target this mechanism to alleviate multiple detrimental aspects of oxygen in the immature lung, especially given that even 21% O_2_ and anything higher is relatively hyperoxia in the context of immaturity.

Compared to adult ASM, [Ca^2+^]_i_ regulation is less well-studied during early development, reflecting lack of age-appropriate cellular models, particularly in humans. Animal models have been extensively used for understanding lung development *per se* (Piedboeuf, 2001; Chinoy, 2003; Land and Wilson, 2005), although the focus has been largely on alveolar growth. We previously showed that ASM cells from lungs of 18–22 week human fetuses represent a good model to understand [Ca^2+^]_i_ in the developing airway given these fASM express a number of intracellular and plasma membrane [Ca^2+^]_i_ regulatory proteins comparable to those in adult ASM, and are responsive to bronchoconstrictor agonists such as histamine (Hartman et al., 2012). The time period of 18–22 weeks also represents a period of rapid bronchial airway growth, and is proximate to survival of prematurely born infants in the neonatal ICU setting where oxygen with assisted ventilation would be implemented as early as 22 weeks gestation.

The CaSR belongs to the Class C family of GPCRs that further include metabotropic GABA-B and glutaminergic receptors and a number of orphan receptors (Brauner-Osborne et al., 2007; Patel et al., 2020). The CaSR, GABA-B and glutaminergic receptors are the most explored in any tissue. Studies in calciotropic tissues such as parathyroid gland, kidney, or bone have shown the CaSR to be critical for sensing [Ca^2+^]_o_ (Brennan et al., 1833; Riccardi and Kemp, 2012; Goltzman and Hendy, 2015) and is targeted with activators (calcimimetics) toward treating hyperparathyroidism, and inhibiting it using negative allosteric modulators (calcilytics) for genetic forms of hypocalcemia (Hannan et al., 2017; Nemeth et al., 2018). CaSR in non-calciotropic tissues is now established, and is pleiotropic in regulating [Ca^2+^]_I_, gene expression, cellular proliferation and production of ECM proteins (Brennan et al., 1833; Riccardi and Kemp, 2012; Conigrave and Ward, 2013; Riccardi et al., 2013; Yarova et al., 2015; Schepelmann et al., 2016). CaSR signaling (as with other Class C GPCRs) can involve multiple G-proteins (G_q/11_ or G_i/o_) (Conigrave and Ward, 2013). *Via* G_q/11_ CaSR can activate the PLCβ-IP_3_ pathway to increase [Ca^2+^]_i_. In addition to intracellular Ca^2+^ release involving IP_3_, CaSR also appears to enhance plasma membrane Ca^2+^ influx, as shown for transient receptor potential canonical (TRPC) channels in pulmonary artery smooth muscle (Smith et al., 2016), and TRPC and SOCE channels in fASM (Roesler et al., 2019). Furthermore, activation of the DAG-PKC pathway or G_i/o_ can further lead to MAP kinase signaling (Kifor et al., 2001) which has implications for longer-term effects relevant to proliferation and ECM production. Here, these parallel pathways of CaSR action may be particularly relevant in developing ASM which have a greater proliferative need in the growing lung, and are likely more dependent on plasma membrane [Ca^2+^]_i_ regulatory pathways. Indeed, we previously showed that CaSR promotes ASM proliferation *via* both PLC (Roesler et al., 2019) and MAPK and ERK1/2 (Yarova et al., 2015), mechanisms that we also explored in the context of hyperoxia in the present study.

Neonatal hyperoxia, i.e., O_2_ higher than 21% is an unfortunately necessary intervention in the context of premature birth, but is known to have significant effects on airway structure and function both in the preterm infants and during subsequent postnatal growth (Britt et al., 2013; Been et al., 2014; Gough et al., 2014; Vollsaeter et al., 2015; Landry et al., 2016). Here, it is important to recognize that given the relative hypoxia under which lung development normally occurs, even 21% O_2_ can be seen as relative hyperoxia at least in the context of immaturity. Conversely, *in vivo* it is likely that sub-epithelial tissues such as ASM experience less than the inspired O_2_ and thus may be under some level of relative hypoxia. Thus, our use of 21% O_2_ was as much based on the “standard” in the field for *in vitro* studies using ASM as well as the need to explore the effects of even greater, clinically relevant levels of O_2_ on the developing ASM. From a technical perspective, in the 50% O_2_ groups, we ensured that hyperoxia was maintained for as long as possible to avoid substantial variations in O_2_ levels that could confound results (i.e., we avoided intermittent normoxia). For immunofluorescence, proliferation, ECM or signaling studies, this was easily accomplished since cells were fixed or otherwise processed immediately following O_2_ exposure. For [Ca^2+^]_i_ imaging, the fura-2 loading was also done in 50% O_2_ for this group, and the imaging protocols were relatively short-duration and thus it was assumed that any reversal of hyperoxia effects were minimal.

While effects on alveolar dysfunction leading to bronchopulmonary dysplasia are better understood, the mechanisms underlying bronchial airway dysfunction and thickening that contribute to wheezing and asthma are less understood. Such reactive airway diseases involve both functional and structural changes with the latter reflected by increased ASM mass (involving changes in cell proliferation and/or size) and increased ECM and thus more fibrotic airways (Reyburn et al., 2012; Martin et al., 2013; Vogel et al., 2015; Vogel et al., 2017a; Prakash, 2016). Mechanisms in the context of inflammation (Hartman et al., 2012), hypercontractility (Faksh et al., 2016), and fibrosis (Vogel et al., 2017) have been explored to some extent. In this regard, there is increasing recognition that O_2_ effects in specific cell types and the types of changes are dependent on extent of exposure structure/function, where with higher levels of O_2_ promote bronchopulmonary dysplasia (Wang et al., 2014; Pabelick et al., 2017), but even moderate hyperoxia can contribute to bronchial changes. For example, at a cellular level, we previously showed that increasing levels of O_2_ result in progressively greater enhancement of [Ca^2+^]_i_ and mitochondrial fragmentation, but interestingly, cell proliferation is increased until >60% O_2_ levels are reached when cell death is predominant (Hartman et al., 2012). Furthermore, we have found that moderate hyperoxia also increases ECM deposition (Vogel et al., 2017). Thus, in the context of airway hyperreactivity and airway thickening, moderate levels of hyperoxia may be particularly relevant. Indeed, the *in vitro* work in human fetal cells is supported by mouse models of neonatal hyperoxia showing increased airway hyperreactivity (Wang et al., 2014; Onugha et al., 2015) and airway collagen deposition (Wang et al., 2014).

The results of the present study now link moderate hyperoxia (50% O_2_) effects on [Ca^2+^]_i_ of developing ASM to the CaSR. Our data first show that 50% O_2_ enhances fASM CaSR expression. Thus, even if there were no additional effects of O_2_, *in vivo* the increased CaSR would allow for greater responsiveness to the higher [Ca^2+^]_o_ of the developing lung and thus enhance [Ca^2+^]_i_ or other CaSR effects. We further show that hyperoxia increases CaSR sensitivity in increasing the amplitude of [Ca^2+^]_i_ response to agonist (but not baseline Ca^2+^), which is suppressed by the negative allosteric modulator NPS2143. In this regard, our data show that hyperoxia enhances intracellular Ca^2+^ release *via* IP_3_ receptor channels (inhibited by Xestospongin C) consistent with a known role for the PLC/IP_3_ pathway in CaSR effects. Therefore, we expected NPS2143 to further Xestospongin C effects, but instead noted alleviation. While the reasons for this effect are not clear, it is possible that the presence of both Xestospongin C and NPS2143 results in compensatory responses of ryanodine receptor or calcium influx activation following histamine stimulation. More nuanced exploration in zero extracellular calcium (thus also limiting CaSR contributions) are necessary to determine such pathways that may or may not involve CaSR.

Consistent with the greater role of plasma membrane pathways in developing ASM, we also find that CaSR contributes to hyperoxia effects on SOCE. We have previously reported that SOCE pathways including STIM1 and Orai1 are present in developing ASM (Hartman et al., 2012) although the mechanisms for such increase are not known. Thus multiple mechanisms (expression, sensitivity, SOCE) exist for CaSR to enhance [Ca^2+^]_i_ in hyperoxia exposed airways.

In the context of [Ca^2+^]_i_ regulation, there is increasing recognition that mitochondria play a key role in Ca^2+^ buffering with further long-term effects on cell structure/function (Prakash et al., 2017). Using rhod-2, we explored mitochondrial Ca^2+^ changes with hyperoxia. Interestingly while mitochondrial Ca^2+^ did not increase with hyperoxia, this was significantly affected/blunted by NPS2143 suggesting that CaSR may be involved in other aspects of mitochondrial function that are not related to oxygen exposure, and that are worth investigating.

Our data also show that CaSR is involved in enhancement of fASM proliferation by 50% O_2_, inhibited by NPS2143. Furthermore, oxygen effects on ECM deposition were blunted by NPS2143, although it is interesting to note that the collagens but not fibronectin are sensitive to NP2143 effects. The mechanisms for these differential effects are unclear, and unlikely to involve pathways such as MAPKs which in adult ASM do affect all three ECM proteins (Freeman et al., 2017). Nonetheless, we do find that at least p42/44 MAPK is increased by 50% O_2_, and sensitive to NPS2143, suggesting a role for CaSR *via* this pathway. Whether this leads downstream to altered ECM is not clear. One potential explanation for differential effects on ECM is upregulation of arginase pathways. Arginase is expressed by airway cells and is considered important in asthma (Maarsingh et al., 2009; van den Berg et al., 2018). We previously showed that arginase is increased in developing airways by hyperoxia and impairs bronchodilation (Ali et al., 2012). The relevance of arginase lies in its downstream product spermine that happens to also be an endogenous CaSR agonist. Thus it is possible that hyperoxia enhances arginases resulting in autocrine effects of spermine. An alternative explanation is the further downstream production of the arginase pathways, namely proline, which is a precursor to collagens (but not fibronectin). Interestingly, we did not find NPS2143 to significantly modulate proliferation or ECM under conditions of normoxia, which we expected if CaSR were to respond to higher [Ca^2+^]_o_ levels for example. The reasons are unclear, but again could be related to lack of upregulated arginase pathways at least for ECM. Of note, we also do not find much effect of NPS2143 on p42/44 MAPK under normoxia conditions, which is different from adult ASM (Yarova et al., 2015).

While the focus of this study was the effect of hyperoxia on ASM, it is interesting to note that hypoxia *per se* induces similar responses in terms of ASM phenotype, such as enhanced airway reactivity and increased smooth muscle thickness (Wang et al., 2018; Noble et al., 2019; Wang and Noble, 2020) at least in a mouse model of intrauterine growth restriction induced by maternal hypoxia. Accordingly, we additionally explored whether HIF1α, known to be involved in hypoxia effects could be a common thread. Here, we used CoCl_2_ to mimic hypoxia and induce HIF1α activation, thus avoiding any confounding roles for ROS which are probably also a common factor between hypoxia and hyperoxia. Interestingly, 50% O_2_ also increased HIF1α activation (reflected by increased nuclear-cytosolic ratio) although our data do not necessarily indicate a role for CaSR in that NPS2143 did not reverse these effects. However, NPS2143 increased HIF1α in hyperoxia only (effects of NPS2143 on HIF1 α nuclear-cytoplasmic ratio were not exhibited in normoxia) and NPS2143 did not reduce HIF1α activation in CoCl_2_, suggesting some linkage between these pathways that remain to be explored further and could be relevant at least to hypoxia-induced changes in the airway, perhaps even during normal development.

Overall, the present study points to potentially important mechanisms by which CaSR inhibition could be used to blunt oxygen effects, particularly in the context of contractility and remodeling that is unlikely to be responsive to current therapies such as bronchodilators or even corticosteroids. Accordingly, future studies need to explore the *in vivo* efficacy of CaSR modulation in alleviating oxygen effects in the developing lung.

## Data Availability Statement

The raw data supporting the conclusions of this article will be made available by the authors, without undue reservation.

## Ethics Statement

The studies involving human participants were reviewed and approved by Mayo Clinic IRB. Written informed consent for participation was not required for this study in accordance with the national legislation and the institutional requirements.

## Author Contributions

AR and JR equally contributed to develop concepts, perform initial experiments, analyze data, and write manuscript drafts. CB, BP, MS, BR, and LN contributed to additional concepts, experiments, and analyses. CP, RM, PM, and YP collaboratively developed concepts and design, interpreted data, and helped with various drafts. YP prepared the final/submitted version that was reviewed and approved by all authors. All authors contributed to the article and approved the submitted version.

## Conflict of Interest

The authors declare that the research was conducted in the absence of any commercial or financial relationships that could be construed as a potential conflict of interest.
